# Detection of Subarachnoid Hemorrhage in Computed Tomography Using Association Rules Mining

**DOI:** 10.1155/2022/1133819

**Published:** 2022-08-31

**Authors:** Hathal Salamah Alwageed

**Affiliations:** College of Computer and Information Science, Jouf University, Sakakah, Saudi Arabia

## Abstract

Subarachnoid hemorrhage (SAH) is one of the serious strokes of cerebrovascular accidents. There is an approx. 15% probability of spontaneous subarachnoid hemorrhage in all acute cerebrovascular accidents (CVAs). Most spontaneous subarachnoid hemorrhages are caused by ruptures of intracranial aneurysms, accounting for about 85% of all occurrences. About 15% of acute cerebrovascular disorders are caused by spontaneous subarachnoid hemorrhage. This illness is mostly caused by brain/spinal arteriovenous malformations, extracranial aneurysms, and hypertension. Computed tomography (CT) scan is the common diagnostic modality to evaluate SAH, but it is very difficult to identify the abnormality. Thus, automatic detection of SAH is required to recognize the early signs and symptoms of SAH and to provide appropriate therapeutic intervention and treatment. In this article, the gray-level cooccurrence matrix (GLCM) is used to extract useful features from CT images. Then, the New Association Classification Frequent Pattern (NCFP-growth) algorithm is applied, which is based on association rules. Then, it is compared with FP-growth methods with association rules and FP-growth methods without association rules. The experimental results indicate that the suggested approach outperforms in terms of classification accuracy. The proposed approach equates to a 95.2% accuracy rate compared to the conventional data mining algorithm.

## 1. Introduction and Background

SAH is a kind of acute cerebrovascular illness that can be caused by a variety of factors. Subarachnoid space can be filled with blood if a blood vessel ruptures in the brain or spinal cord. The most frequent cause is cerebral thrombosis. A subarachnoid hemorrhage can be classified into two groups according to the cause: spontaneous and traumatic. About 15% of acute cerebrovascular disorders are caused by spontaneous subarachnoid hemorrhage. This illness is most caused by brain/spinal arteriovenous malformations, extracranial aneurysms, and hypertension. This syndrome can be caused by a variety of conditions, such as Moyamoya disease, vasculitis of the brain, malignant tumors, homological abnormalities, meningitis, encephalitis, and anticoagulation therapy issues [1, 2]. The most common subarachnoid hemorrhage occurs when an intracranial aneurysm ruptures or a vascular abnormality rupture occurs, while the others are uncommon. Generalized nonaneurysmal nonperimesencephalic subarachnoid hemorrhages (PNSHs) and atraumatic convex subarachnoid hemorrhages (cSAHs) are the most common types of spontaneous subarachnoid hemorrhages. Rinkei offered the following concept of PNSH in 1991: the front of the midbrain is where the central section of the subarachnoid hemorrhage occurs. The bleeding might be accompanied by an enlargement of the annular cistern base. The longitudinal fissure cistern's front half is not entirely filled. Neither a cerebral hemorrhage nor an expansion of the lateral fissure cistern is apparent [3]. Approximately 15% of all spontaneous subarachnoid hemorrhages, according to some estimates [4], are brought on by PNSH. sSAH occurs in the sulcus gyrus of the superficial cerebral cortex. The cerebellum's surrounding big sections, the anterior and posterior longitudinal fissures, or any subcutaneous cisterns are not typically affected by bleeding [5], and only 7.45 percent of the time [6] is the ventricle affected. As a result, early identification and treatment are crucial in preventing and treating subarachnoid hemorrhage complications. The most common kind of spontaneous subarachnoid hemorrhage is intracranial aneurysmal subarachnoid hemorrhage, which accounts for around 85% of cases [7]. Intracranial aneurysm rupture is a gradual process. The mortality rate of the first hemorrhage might reach 40%, and the disability rate can reach 33% [8, 9]. The mortality incidence of rerupture is as high as 60–70% if it is not recognized and treated promptly, and the damage is significant [10]. More than 60 million people have aneurysms, and each year over 200,000 people have a ruptured and hemorrhaged brain aneurysm, posing a serious risk to their lives and health and substantial financial and psychological costs to society. Therefore, early detection can be achieved with appropriate methods.

Intracranial aneurysms must be extracted, and appropriate therapeutic therapies must be carried out. Since its inception, data mining has attracted a growing amount of interest. It works well with large, incomplete, and noisy practical applications. Data mining is used to analyze data that has hidden value. The data's potential information is discovered through induction, generalization, and reasoning. Simultaneously, the data are continually enhanced through the mining process, allowing the data to be completely gathered, interpreted, and utilized to its full potential [11, 12]. New technologies are influencing medicine and research in related sectors. We cannot fully utilize and benefit from data until we store and analyze massive amounts of data. Due to the inability of most modern database systems to process large quantities of data, these data cannot be used for disease analysis, diagnosis, and pathological research. Data expertise cannot predict how data will develop in the future. In medical data mining, doctors can gather precise information about illnesses and how they are treated, improving hospital management. Data mining and knowledge discovery are discussed in this article, as well as how data mining is used in medical applications. The fundamental theories, broad structure, and primary technologies and methodologies of data mining are all thoroughly explained, followed by an examination of the unique characteristics of medical data. And then mix the two to come up with a process model for medical data mining. Using association rule extraction and its application to medical image mining, photographs are merged using association rule extraction using the connected theories of association rules and the essential methods of association rule extraction. The texture features of subarachnoid hemorrhage in CT images are extracted using a gray-level symbiosis matrix in order to provide supplemental classification and diagnosis. Computed tomography (CT) scan is the common diagnostic modality to evaluate SAH, but it is very difficult to identify an abnormality. Thus, automatic detection of SAH is required to recognize the early signs and symptoms of SAH and to provide appropriate therapeutic intervention and treatment. In this article, the gray-level cooccurrence matrix (GLCM) is used to extract useful features from CT images. Then NCFP-growth algorithm is applied, which is based on association rules. Then, it is compared with FP-growth methods with association rules and FP-growth methods without association rules. The experimental results indicate that the suggested approach outperforms in terms of classification accuracy. Further, the article is divided into the following sections: Section 2 describes the literature review, Section 3 is the proposed methodology, Section 4 is the experimental analysis, and Section 5 is the conclusion.

### 1.1. Contributions

Contributions to this research paper are as follows:

- A machine learning technique for detecting subarachnoid hemorrhages in CT using association rule mining is proposed.
- The GLCM is used to extract useful features from CT images.
- The accuracy rate is improved up to 95.2%.

## 2. Literature Review

A perimesencephalic nonaneurysmal subarachnoid hemorrhage (PNSH) contributes to approximately 21%– 68% of all spontaneous subarachnoid hemorrhages with negative DSA. Most patients with this disease are asymptomatic, have a small bleeding site, have a successful treatment process, and are found to have no complications, such as vasospasm or hydrocephalus, and recurrences are limited. Compared to aneurysmal subarachnoid hemorrhage, cavity hemorrhage has dramatically different consequences. It is considered separate from benign subarachnoid hemorrhage. The literature argues that for a condition with low morbidity, death, and disability rates, it is relevant for complications induced by examination procedures to be fewer than 0.5 percent. As a result, the technique of examination we use should be noninvasive and effective. Not only does it decrease the patient's exposure to the inspection procedure, but it also has greater sensitivity, allowing it to rule out aneurysms with a high death and disability rate. To effectively diagnose PNSH, it must also have a larger negative predictive value. In addition, another major subtype of subarachnoid hemorrhage, spontaneous localized subarachnoid hemorrhage, was found this year. Only a few cases of cSAH had been recorded before Spitzer et al. described 12 cases in 2005 [5]. Kumar et al. found that it occurs in roughly 7.45 percent of people [6]. In addition, there has been no extensive study of its incidence and gender differences. It has a different cause of bleeding than PNSH, but it has a good prognosis and is categorized as a benign subarachnoid hemorrhage. Aneurysms should be recognized from such subarachnoid hemorrhages in terms of diagnosis and therapy. Digital subtraction angiography (DSA) is used to diagnose aneurysms, assess their preoperative status, and determine other vascular imaging indicators [12, 16]. DSA, on the other hand, has a complication risk of around 1-2 percent, with about 0.5 percent experiencing irreversible neurological impairment. It can be fatal in extreme circumstances. Other invasive treatments, such as angiography, are available. The drug's drawbacks, such as radiation danger, long inspection times, and high costs, limit its widespread use in intracranial aneurysm screening and follow-up observation [17, 18]. It is particularly inappropriate for exclusion screening and follow-up of benign SAH such as PNSH. As a result, noninvasive examination approaches are gaining popularity. In modern clinics, CT angiography has become widely used to detect intracranial aneurysms, treat endovascular problems, and perform surgical procedures instead of using only DSA technology. These noninvasive vascular imaging approaches allow for the accurate diagnosis of aneurysms while eliminating the risks associated with cerebral angiography [17, 18].

Manual CT image detection, on the other hand, has low accuracy and efficiency, and the technology of data mining is helping to alleviate this problem. Image mining is a young field that has just recently evolved. Among the areas involved are computer vision, image processing, image retrieval, data mining, machine learning, database, and artificial intelligence. Image mining is still in the exploratory research stage, even though these disciplines are quite established in their respective domains. Many researchers, both at home and abroad, have actively explored this discipline and made significant efforts in the following areas:

1. Celestial image mining: this technology uses scientists' precisely classified sky photographs as the training set to create a model for detecting galaxies, and it has been used to effectively discover volcanoes on Venus [19].
2. Satellite remote sensing image mining [20]: satellite imagery is being used more frequently in several fields to solve surface challenges through remote sensing. When moving targets are detected in remote sensing photographs and stored with their original images in a database, a whole host of additional information can be retrieved, including connections to the moving targets.
3. Spatial data mining [21]: using this tool, it is possible to understand geography, identify spatial correlations, and establish links between spatial data and nonspatial data. People are hoping to build a geographic data cube and mine spatial data using it. In this subject, geographical data association analysis is a prominent research issue, and various methods have been presented. An image data mining software prototype developed at NASA's Jet Propulsion Laboratory, “Diamond Eye,” automatically extracts semantic information from a picture and determines the topography of craters. Satellite detection and analysis have been very useful [22].
4. Medical image mining [23]: image mining systems have benefited from the availability of many medical photos. The physician has always documented the diagnosis along with medical images. Visual qualities in medical imaging may be connected to diagnosis data in a variety of ways. Medical imaging has become a specialized field. Some research organizations, for example, investigate the gap between damaged brain tissues. Some research organizations utilize similar approaches for judging early breast cancer [24, 25]. The diagnostic record's correlation between features and pathological characteristics can aid doctors in determining the tumor's location. Multimedia Miner is a prototype application developed by Simon Frase University in 1998. In the development of this approach, the DB-Miner relational database mining system and C-BIRD were used [26]. By utilizing multidimensional analysis technologies, a multimedia cube management system is capable of building multimedia data cubes for a variety of purposes, including summary knowledge for categorization and association rule knowledge for association purposes. MM-Associator is one of the modules, and it mostly mines image association rules. Image size, color, and image description are among the data linked by these rules [27]. The prototype system is made up of three functional modules: this module describes multimedia data attributes using several abstract layers while allowing users to scroll up and down to examine data on different levels. (2) You can search for association rules using image or video data with MM-Associator. (3) MM-Classifier classifies multimedia materials and provides explanations for each class, according to Chen et al. [28]. Machine learning-based algorithms play a vital role in lowering fatality rates and effectively managing bleeding. An IoT classification system based on support vector machines and feed-forward networks is presented. The machine learning-based tool can help specialists diagnose and manage brain hemorrhages by providing information on the kind of hemorrhage. Wang et al. [29] gave an overview of how deep learning algorithms may be used to identify and classify bleeding in CT images automatically. Using AI-based technologies to automate the diagnostic process would eventually lead to a more effective and more timely cure. To detect bleeding accurately, they use a deep learning algorithm based on CNNs.

At this time, image mining research is rather developed, and it can do different processing on medical pictures, allowing difficult-to-observe lesions to become clearer while also providing a degree of auxiliary diagnosis. Using this technology, hospitals are able to communicate better with each other, diagnose and treat illnesses more efficiently, and reduce their workload on equipment, creating an improvement in overall medical quality.

## 3. Methodology

### 3.1. Association Rule's Overview

The link between distinct things that exist in the same event is referred to as association rules. Agrawal et al. suggested the extraction of association rules for the first time in 1993. Since its development in the 1990s, it has evolved into one of the most important data mining methods. On the one hand, mining association rules can provide us with association linkages at multiple conceptual levels. The association rule mining technique can be applied to obtain association rules for laws between the different levels of a hierarchical tree describing a domain-related idea. Alternatively, various types of data sets have unique association rules. The associations returned by association analysis are evaluated by two indicators: the level of support and the level of confidence represents both the level of interest the community has in the rule and its reliability. Finding the rules that have higher support and confidence than the threshold rules with the least support and confidence is the goal of association rule mining. However, there may be times when low-support limitations are required, such as during sickness surveillance. Association rule mining usually consists of two steps: finding data points that meet the minimum degree of support, followed by finding frequently occurring data points. A strong association rule is constructed by taking frequent data points with the lowest confidence level as the second step. The first phase of this mining strategy, which involves successfully locating frequent data points, is the most difficult because it has the biggest effect on the algorithm's performance. These two-step mining procedures are used by many famous algorithms, such as Apriori, DHP, and others, for mining association rules.

### 3.2. Classification of Association Rules

1. Boolean association rules and numerical association rules are classified according to the type of variables they are associated with. An association between a quantitative item or attribute is specified by a quantitative association rule. A Boolean association rule is composed of items that are either present or absent.
2. It is separated into single-dimensional association rules and multidimensional association rules based on the dimensionality of the data included in the rules. A one-dimensional association rule deals with some associations in a single attribute and has just one dimension for each item or attribute. A multidimensional association rule is one that deals with the relationship between two or more unique attributes and has two or more dimensions.
3. Each association rule can be categorized into a single-level rule and a multilevel rule based on the level of abstraction from the data included in the rule. There are no objects or characteristics in a rule collection whose association is based on a single abstraction level. Items or characteristics from distinct abstract levels are involved in the multilayer association rules.

### 3.3. Association Rules Process

“Association rule mining” is the process of removing rules from a transaction database that satisfy the minimal support and confidence conditions. A more basic mining strategy is to calculate all viable rules, a and b. This tactic, however, is obviously ineffective. A little data collection can provide hundreds of rules. More than 80% of the rules could be removed if the minimal support level and the confidence level were decreased to 25% and 50%, respectively. As a result, the rules must be pruned first to increase mining efficiency. The calculation method for the rule's support shows that the rule's *X*⟶*Y* support is solely dependent on the support count of the item set {*X* ∪ *Y*}. A significant number of association rule extraction algorithms are divided into two stages: finding common data points and finding association rules. The former is used to identify which data points people are interested in (i.e., those that obtain more support than a pre-determined support threshold), also known as frequent data points. These rules are derived from frequently occurring sets of items with confidence levels greater than the threshold.

### 3.4. Algorithm for Association Rule Mining

#### 3.4.1. Apriori Algorithm

The Apriori approach [30], developed by Professor Agrawal for analyzing shopping basket data, is the most basic and extensively used algorithm in the study of association rules. By scanning the database several times, the method obtains single-layer Boolean association rules that require frequent data points from the Latin Apriori, which means “from the beginning.” Recursion, which is based on frequency set theory, is an important idea. Prior knowledge of often recurring data points gives the Apriori algorithm its name. It mines frequent data points using a circular hierarchical search. This loop generates (*k* + 1)-data points using *k*-data points. The most common 1-items set is incredibly *T*1, and *T*1 is then used to mine *T*2, and *T*2 is then used to mine *T*3. This process must be repeated for each layer of mining, up to the point where no more frequent items can be mined. *T*1 is generated by the algorithm, which then searches the database *D*, removes a portion of the item set from the candidate set, and returns *T*2. It then scans the database *D* again, generating a candidate set of *T*3 through *T*2. This step is continued until there are no more data points to add to the list. The Apriori algorithm has a unique feature. In compliance with the definition, an itemset cannot be a frequent itemset if it does not meet the minimal support condition. A new itemset aI will be created from the itemset I if a new item is added to it. I cannot be a frequent itemset since the number of occurrences must be less than I. Its converse statement is that if an item set fails to reach the minimal support level, then all feasible supersets fail as well. Following this, we describe how Tk designs Tk + 1 and uses Apriori characteristics to identify frequent data points through the two procedures of joining and deleting.

1. Connection step: to mine Tk + 1, two Tk itemsets can be joined to form a candidate set of Tk + 1, which is designated as Lk + 1. The two data points in Tk are *t*1 and *t*2. If the data points *t*1 and *t*2 are identical except for the final and penultimate items, other items are identical; however, the penultimate item is not identical. As long as the entry in each Tk's *t*1 and *t*2 is expected to be in lexicographical order, then Tk's *t*1 and *t*2 can be connected, and this connection method can ensure the presence of all and nonduplicate candidate item sets.
2. Deletion step: The above connection principle generates the candidate set Lk + 1, which is a superset of Tk + 1. There are two parts to the deleting procedure here as well. First, according to the Apriori property, eliminate all previously discovered supersets of infrequent data points. Then scan the database for data points with support less than the minimal support criterion, and mark them as infrequent data points.
3. We may deduce from the deletion step that each item put in Lk + 1 has to be searched in the database before being added to Tk + 1. The Apriori algorithm's verification step is its bottleneck. In the example above, the database would need to be searched ten times, resulting in a substantial I/O load, and here the Apriori algorithm would gain most of its benefits. Following the discovery of all frequent data points, association rules must be generated using frequent data points. Most algorithms, including Apriori, use a similar rule generating mechanism. All nonempty subsets *S* are constructed for each frequent itemset *T*. For each nonempty subset, if *σ*(*T*)/*σ*(*S*) ≥ *min* _conf, a strong association rule *S*⟶(*T* − *S*) is generated.

The general form of the Apriori Algorithm [30] is as follows:

 
*L*_1_ = {large 1-data points}
  For (*k* = 2; Lk-1 ≠ ≠0; *k*++) do
  Ck = Apriori-gen (Lk − 1)
   for (all transactions *t* *ϵ* Ct, do
    increment c.count
  end
   Lk = (*c* *ϵ* Ck | *c*.count ≥ minsup}
    End
  Solution = Uk L_k_

#### 3.4.2. DHP Algorithm

By interlinking frequent data points of the previous layer, frequent data points of the next layer can be linked to the next layer's candidate sets. After obtaining the frequent item sets of this layer from the candidate set, the cycle continues. As the candidate set grows, the screening algorithm becomes less efficient since it requires one-to-one comparisons with the database to compute the level of support for each item and reduce the number of candidate sets to a minimum, thus decreasing the number of comparisons, so that algorithmic speed can be improved. Using DHP (Direct Hash Table Prulling), you can reduce the number of superfluous candidate sets for a mining association rule through the use of a hash table structure. When building (*k* + 1)-data points from k-data points, create a hash bucket that will be used later to further filter the set of candidates. At the same time, the database needs to regularly be updated with fresh data points. Although resources may be needed to update the hash bucket and a database, the DHP technique dramatically lowers the number of candidate sets, improves performance, and minimizes comparisons.

#### 3.4.3. Algorithm for Partition

Apriori uses the Partition algorithm to generate rules from frequent data points. On the other hand, the Partition technique segments the database to reduce the cost of each database scan. The essential premise is that if an item set is common over the entire database, it must also be frequent across a subset. A frequent itemset mining operation is performed on each segment of the database in order to determine the frequent itemset for that segment. The second stage involves combining all the segmented frequent item sets into a large candidate set, then filtering and verifying the true frequent item set of the whole database by comparing the large candidate set to the entire database. Because the Partition method uses the divide-and-conquer strategy, the entire procedure only searches the database twice, considerably lowering I/O usage. However, sorting the database before mining is required to eliminate duplicate frequent data points between various segments, which restricts the implementation of the Partition method to some extent.

#### 3.4.4. Algorithm for FP-Growth

The FP-growth method may immediately build frequent data points without going through the candidate item set stage. The FP-growth algorithm likewise employs the divide-and-conquer technique, but it does so in two stages: after compressing the entire database into an FP-tree while maintaining item information, a series of condition databases corresponding to frequently occurring items are generated for the compressed database. The FP-growth method is the most distinct from the other modified Apriori algorithms discussed. They have distinct classification standards for common data points. FP-growth classifies frequent items in decreasing order of support, whereas the Apriori method divides frequent items into 1-item sets, 2-item sets,...., and *k*-item sets according to their size; item mining is done in increasing order of length, whereas item mining is done in decreasing order of length. After sorting, the transaction's frequent elements are added to the FP-tree, which is then mined recursively.

#### 3.4.5. NCFP-Growth Algorithm

**Figure 1:**
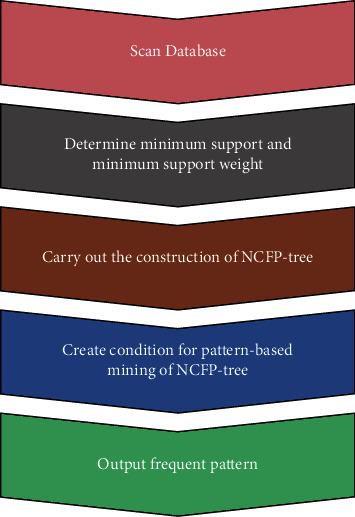
NCFP-growth algorithm.

The shortcomings of mining association standards have been repeatedly mentioned since the topic was first raised. Several additional criteria have been proposed to improve the analysis of association rules and avoid the development of fake association rules. In this article, the NCFP-growth algorithm is proposed as an improved frequent pattern tree construction based on the NCFP-growth algorithm. By effectively incorporating interest degree weights into the system, the FP-growth algorithm reduces the number of redundant and inaccurate rules. Furthermore, this strategy effectively compresses the search space of the algorithm and reduces the size of the tree and the system storage space compared to the FP-growth method. The NCFP-construction tree: the transaction database DB is the input; min sup is the minimal support threshold; min up is the minimum interest weight; min up is the whole collection of frequent patterns. This is how it's done: (1) examine the DB (transaction database); (2) identify the W elements with the highest degree of support using the minimal support minimum support; (3) arrange the *W* elements in table *L* in ascending order of degree of support. (4) Under NCFP-tree, create a root node and set its value to null. (5) Complete each transaction using the transaction database. (6) Sort the frequent things in each transaction that satisfy min up in the order in *L*; the sorted table is labelled [*p*|*P*, *T*], where *p* is the first element and *P* is the list of remaining elements. (7) If *T* has a child named *N* item name = *p* item name, *N*'s count is incremented by one; otherwise, a new node *N* with a count of one and a link to its parent node *T* is created. Utilizing the structure of a node chain, connect *N*'s node chain to the node with the same item name; if *P* is not empty, repeat steps 2 and 3 above. Afterwards, the NCFP-tree is built in the same manner as the FP-tree. As can be seen in Figure 1, the NCFP-growth algorithm is illustrated.

NCFP-growth algorithm shows that the system filters the original frequent items further when a new threshold is applied to them, thus reducing the possibility that too many absurd or redundant associations are generated, and enabling users to extract more practical association rules that are relevant to their needs.

### 3.5. Gray-Level Cooccurrence Image's Matrix

Each pixel in each image has a distinct or same gray level. The texture information in an image can be analyzed by considering the distance between two pixels. The conditional probability density function of approximated gray levels can be used to determine the gray levels of two pixels based on their cooccurrence matrix. To calculate the probability of another pixel with a gray level of j at a distance (*D*_*x*_, *D*_*y*_) having the same gray level as the pixel with a gray level of *I*, we use the following formula:

(1) $$\begin{array}{c} P\left(i,j,d,\theta \right)=\left\{\left(x,y\right)f\left(x,y\right)=i,f\left(x+D_{x},y+D_{y}\right)=j,x,y=0,1,2,\ldots ,N-1\right\}\text{.} \end{array}$$

There are four values: 0°, 45°, 90°, and 135° for the gray level, *x*, *y* for the coordinates of the image pixels, and for the gray level. Thus, the gray-level pair (*i*, *j*) describes the texture information of the image. Clearly, the matrix resulting from the gray-level cooccurrence is symmetric. If, for example, *M*(1,1)=1, which means that there are only two horizontally adjacent gray level 1 pixels in the original image, then *M* (1,1) = 1. Grayscale pixels with gray scales 1 and 2 are horizontally adjacent in the original image. Therefore, *M*(1, 2)=2, meaning there are two grayscale pixels.

The image's coordinates are given by *x*, *y* image's *θ* gray level as indicated by *i*, *j* and the image's direction is expressed by four values: 0°, 45°, 90°, and 135°. The gray-level pair describes the texture information of the image in this way (*i*, *j*). The GLCM that results is obviously symmetric. Using the GLCM element *M*(1,1) as an example, *M*(1,1)=1 indicates that in the original picture, only a pair of pixels with a gray level of 1 are horizontally contiguous. Because there are two pairs of pixels in the original image with gray scales of 1 and 2 horizontally contiguous, *M*(1, 2)=2,

### 3.6. Association Rule Mining Method

**Figure 2:**
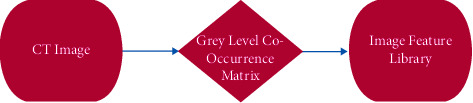
Process model of CT image mining.

Thus, by converting gray-level cooccurrences from the arachnoid CT images into mathematical texture features, the process model of CT image mining is shown in Figure 2.

Image cropping, image noise reduction, and image enhancement are all required before extracting features. After the CT is complete, you can create a matrix of gray-level cooccurrences. The main goal of creating the matrix is to figure out what the gray level L and step are. Adding up the amount of data and computing the GLCM of the image determine two parameters of long *D*. Let's use the 288288 image format as an example. When the image is not compressed and step size *D* = 1 and gray level *L* = 256 is used, calculating the GLCM will take a very long time. In order to compress the original image, *L* was reduced and *D* was raised. With smaller gray levels *L* and larger step sizes *D*, the GLCM is more affordable to construct, but the less accurate the results, the more information lost. The higher the cost of building the gray-level cooccurrence matrix, the higher the *L*, the smaller the *D*, and the more information kept. By identifying *L* and *D*, one can create the image's gray-level cooccurrence matrix. The matrix can be retrieved for its six attributes, which are energy, contrast, entropy, median, local stability, and correlation. A CT image mining database may be created by combining the following 6 elements of the arachnoid CT picture with the doctor's pre-diagnosis of the patient's subarachnoid hemorrhage (abbreviated as PD). PN, PD, *H*, *I*, *J*, *K*, *L*, *M*, and Class are the attributes for each instance of each object stored in the database. PN indicates a probable diagnosis, PD indicates the physician's prediagnosis, *H* indicates the characteristics extracted from six gray-level cooccurrence matrices, *K*, *L*, and *M* indicate whether the subarachnoid hemorrhage diagnosis is ultimately confirmed, and Class indicates the actual statement made by the physician. Using association rule mining, it is possible to diagnose and classify a database that has already been created. Here is a demonstration of how to mine a database section for subarachnoid hemorrhage diagnostic criteria. The present database is included in Table 1.

The following recommendations are based on clinical medical experience and intelligent image diagnosis principles; subarachnoid hemorrhage, or Class 1, is defined as a diagnosis that is based first on a clinical diagnosis and then on arachnoid CT image values that are at least 1, and at least two of the *K*, *L*, and *M* values are 1. According to the study, the patient still suffered from subarachnoid hemorrhage, even though the initial diagnosis of No. 81 in the database did not match the initial criteria. This demonstrates that mining association guidelines should be used to assess the practicality and scientificity of diagnostic criteria.

## 4. Experiment and Analysis

### 4.1. Data Collection

There are several subarachnoid hemorrhage findings in CT images that appear to have irregular edges, dark surfaces, rough textures, and uneven grayscale distributions.  Table 2 represents the Quantity and Grouping of Data image, which has 309 groups with a total of 642 images in each group. We assembled 50 sets of 1000 arachnoid CT images (both normal and pathological) from an affiliated hospital of a top-three medical hospital into an image database to explore how arachnoid CT images are applied clinically.

### 4.2. NCFP-Growth Compared to Other Growth Algorithms

#### 4.2.1. UCI Data Experiment Using Algorithms

**Figure 3:**
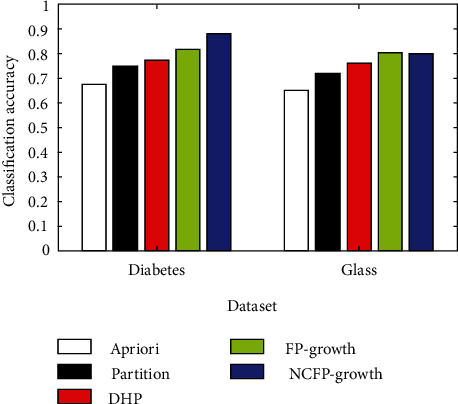
Comparison of five algorithms for classifying glass and diabetes.

**Figure 4:**
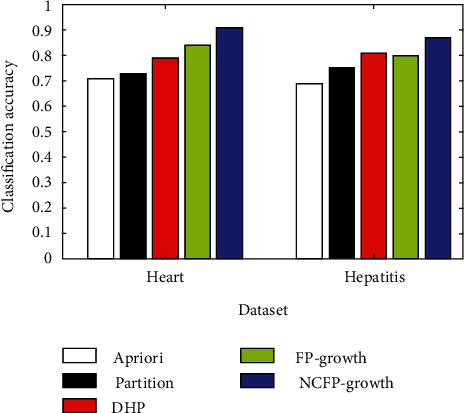
Hepatitis and heart disease: accuracy of five algorithms.

**Figure 5:**
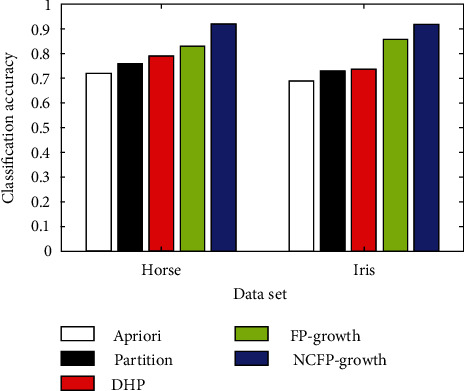
Five algorithms for iris and horse classification accuracy.

**Figure 6:**
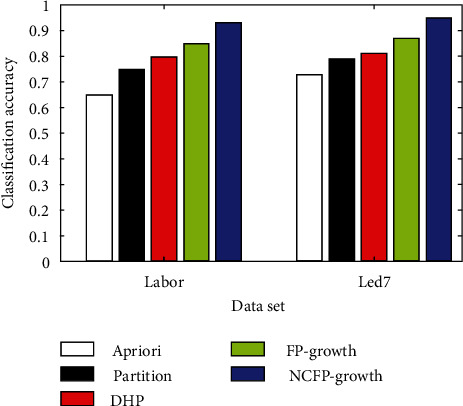
Five algorithms used to classify led7 and labour in accuracy.

The NCFP-growth algorithm put forward in this study is among a number of algorithms that are assessed using common data from the data mining industry. In its ML Repository, UCI has eight datasets: diabetes, glass, hepatitis, iris, horses, labour, and led7.  Figure 3 shows the comparison of five algorithms for classifying glass and diabetes, Figure 4 shows hepatitis and heart disease and the accuracy of five algorithms, Figure 5 shows the five algorithms for iris and horse classification accuracy, and Figure 6 shows the five algorithms used to classify led7 and labour in accuracy. We illustrate how to achieve comparable outcomes in comparison experiments using the five strategies outlined above.

The histogram clearly illustrates that the NCFP-growth algorithm outperforms the others in terms of accuracy. Due to the NCFP-growth algorithm, the mining accuracy is improved. Since it trims duplicate candidate's sets while searching for frequent item sets, the Partition algorithm's divide-and-conquer strategy can considerably increase the algorithm's performance.

#### 4.2.2. Analyzing the Subarachnoid Hemorrhage Dataset to Experiment with NCFP-Growth

**Figure 7:**
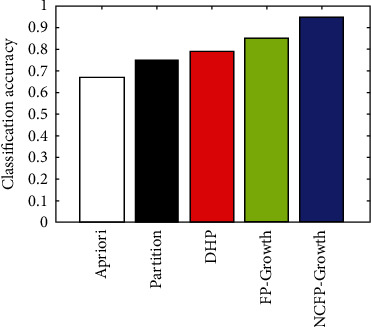
NCFP-growth classification accuracy and other algorithms.

On the subarachnoid hemorrhage data set, we perform 10-fold cross-validation and compare the classification accuracy with those of the different standard approaches discussed in the section, including Apriori, DHP, Partition, and FP-growth, which is depicted graphically in Figure 7. Experimental settings include 1% minimum support and 50% minimum constraint.  Table 3 represents the number of FP-growth and association rules of NCFP-growth as follows.

Table 3 illustrates the fact that NCFP-growth is less complex than other algorithms, with 9252 candidate association rules instead of 3122 in FP-growth. NCFP-growth also reduced its classification rules from 63 to just 26 compared to FP-growth. Figure 7 illustrates that using the NCFP-growth algorithm equates to a 95.2% accuracy rate compared to the conventional data mining algorithm.

## 5. Conclusion

Data mining for medical purposes uses association rules extraction technologies to examine the application of these technologies. This article employs association rules mining to diagnose subarachnoid hemorrhage (SAH). The article explains in detail the approach used to implement this data mining technique. An Apriori algorithm for identifying frequent associations is presented, along with a review of the principles and fundamentals of association rule extraction. GLCM is the most powerful feature extraction used for characteristics of CT scans, followed by association rule image mining. Moreover, alternative mining methods based on different growth algorithms are compared to the NCFP-growth algorithm based on association rules. The NCFP-growth algorithm was found to be more accurate for SAH and it might be used to diagnose actual cases of such disease. The process segmentation is not applied in the proposed model, which is the main drawback of this article. Additionally, the ensemble of deep learning models will be applied in the future for the diagnosis of SAH. The experimental results indicate that the suggested approach outperforms in terms of classification accuracy. The proposed approach equates to a 95.2% accuracy rate compared to the conventional data mining algorithm.

## Figures and Tables

**Table 1:** CT image feature data fragment.

PN	PD	*H*	*I*	*J*	*K*	*L*	*M*	Class
60	1	1	0	0	1	0	1	1
61	1	1	0	1	0	1	0	0
62	1	1	1	1	0	1	1	1
63	0	0	1	1	1	1	0	0
64	1	1	0	0	1	0	1	1
65	0	0	1	0	1	0	1	0
66	1	1	1	0	1	1	0	1
6667	1	1	1	1	0	1	0	1
68	1	0	1	1	1	1	0	1
69	0	1	0	0	0	1	1	0

**Table 2:** Quantity and grouping of the data image.

Classification type	Group	Number of images
Normal	50	900
Abnormal	15	300

**Table 3:** Number of FP-growth and association rules of NCFP-growth.

Algorithm	Number of CARs	Number of classifiers
FP-growth	8756	63
NCFP-growth	3122	26

## Data Availability

The data used to support the findings of this study are included within the article.
